# Risk of hepatitis B virus reactivation in people with multiple sclerosis treated with ocrelizumab: an observational study from Turkey

**DOI:** 10.1007/s00415-024-12333-0

**Published:** 2024-04-05

**Authors:** Muammer Çelik, Cavid Baba, Çağlar Irmak, Serkan Özakbaş, Vildan Avkan-Oğuz

**Affiliations:** 1 Faculty of Medicine, Department of Infectious Diseases and Clinical Microbiology, Dokuz Eylul University, 35330 Izmir, Turkey; 2 Dokuz Eylul University, Graduate School of Health Sciences and Urla State Hospital, Izmir, Turkey; 3 Faculty of Medicine, Department of Neurology, Izmir University of Economics, Izmir, Turkey

**Keywords:** Multiple sclerosis, Ocrelizumab, Hepatitis B, Antiviral prophylaxis, Hepatitis B virus reactivation

## Abstract

**Background:**

The risk of hepatitis B virus (HBV) reactivation remains unclear in people with multiple sclerosis (MS) receiving ocrelizumab. We aimed to assess HBV seroprevalence and reactivation risk in MS patients on ocrelizumab and to evaluate the effectiveness of antiviral prophylaxis against HBV reactivation.

**Methods:**

In this single-center, cross-sectional study, 400 people with MS receiving ocrelizumab were screened for HBV at baseline and antiviral prophylaxis was implemented based on serological results. Patients were monitored for HBV reactivation, and outcomes were analyzed.

**Results:**

Among 56 (14%) patients who had serology compatible with occult or resolved HBV infection, 49 (85.7%) received antiviral prophylaxis regularly and had no HBV reactivation during the follow-up. Reactivation of HBV occurred in 2 out of 7 (28.6%) patients who did not receive antiviral prophylaxis and in one patient who did not adhere to the prophylaxis regimen. All patients with reactivation had anti-HBs levels below 100 mIU/mL and the median titer was significantly lower than the patients with no HBV reactivation (*p* = 0.034).

**Conclusion:**

This study highlights a 14% anti-HBc positivity, indicating a potential risk for HBV reactivation in people with MS receiving ocrelizumab. This suggests the importance of vigilant monitoring and the implementation of prophylactic measures. Our recommendation emphasizes antiviral prophylaxis, particularly for patients with low anti-HBs, and a pre-emptive strategy for others.

## Introduction

Multiple sclerosis (MS) is an immune-mediated disease affecting the central nervous system and is characterized by demyelination and/or neurodegeneration. Globally, the estimated number of people with MS reached 2.8 million in 2020, which is 30% higher than in 2013. The 2020 global prevalence is 35.9 per 100,000 people [1]. According to the Atlas of MS 3rd Edition, 70 individuals per 100,000 inhabitants are suffering from MS in Turkey [2].

Disease-modifying therapies (DMTs) used for MS treatment possess immunomodulatory or immunosuppressive effects. Ocrelizumab is an anti-CD20 monoclonal antibody for the treatment of primary progressive and relapsing–remitting MS. Due to the homology of ocrelizumab with other B-cell-targeting DMTs, such as rituximab, the possibility of hepatitis B virus (HBV) reactivation is acknowledged [3]. B-cell depleting drugs may elevate the risk of HBV reactivation in both patients with chronic hepatitis B and those with past resolved HBV infection [Hepatitis B surface antigen (HBsAg)-negative, anti-hepatitis B core (HBc) total positive, with or without anti-HBs positivity] [4, 5].

There are limited studies evaluating the risk of HBV reactivation in people with a diagnosis of MS receiving ocrelizumab. In the phase III trials of ocrelizumab in people with MS, those with positive HBsAg were excluded, and individuals with a history of past HBV infection were monitored for HBV DNA levels, with no observed instances of HBV reactivation [6, 7]. However, a case has been reported in the literature where a MS patient with a history of past HBV infection experienced HBV reactivation associated with the use of ocrelizumab [8].

The risk of HBV reactivation in people with MS receiving ocrelizumab still remains an unresolved issue. According to the current guidelines of the Asian Pacific Association for the Study of Liver (APASL), ocrelizumab is categorized as uncertain in the risk stratification table for HBV reactivation. The APASL guidelines state that there is no prophylaxis recommendation until further evidence is available [9]. While there are no specific recommendations for HBV prophylaxis in people with MS receiving ocrelizumab in national and international guidelines, prophylaxis is planned based on recommendations made for anti-CD20 therapies used in other indications [10–13].

HBV indeed continues to pose a significant public health threat worldwide, particularly in lower- and middle-income countries. In a previously published report by the World Health Organization (WHO), it was estimated that 296 million individuals globally were living with hepatitis B infection and an estimated 90% of hepatitis B infections remain undiagnosed [14]. There is limited research evaluating the burden of HBV disease and HBV reactivation in people with MS receiving ocrelizumab in Turkey, which is a middle-income country with moderate HBV endemicity, and also worldwide [15].

In this study, our objective was to assess the prevalence of HBV and the risk of HBV reactivation in people with MS receiving ocrelizumab treatment. Additionally, we aimed to evaluate the effectiveness of antiviral prophylaxis in preventing reactivation specifically in individuals with resolved HBV infection.

## Materials and methods

The patient population participating in the study is derived from a cohort that includes over 3600 individuals with MS, with half from Izmir and the other half from various regions of Turkey. Therefore, there is a high likelihood of representing Turkey extensively.

In this single-center, cross-sectional study, we enrolled all patients aged 18 and older who have received at least one dose of ocrelizumab for the treatment of MS, between June 2018 and February 2023. Patients receiving immunosuppressive or immunomodulatory treatments other than ocrelizumab (excluding pulse steroid therapy) and with missing data were excluded from the study. Demographic information such as age, gender, and residence, along with clinical data, HBV screening results, and other laboratory findings, was obtained from the hospital information management system or e-Nabız (the personal health record system of the Turkish Ministry of Health). The patients were prospectively monitored; however, data collection was conducted retrospectively.

At our facility, prior to ocrelizumab treatment, each patient is screened for HBV using HBsAg, anti-HBc total, and anti-HBs, routinely. With their HBV screening results, the patients are referred to the outpatient clinic of the Department of Infectious Diseases and Clinical Microbiology. Patients are divided into two groups based on their serological test results and subsequently monitored accordingly.

1. HBsAg positive: For the baseline assessment, liver function tests (LFTs) including aspartate aminotransferase (AST), alanine aminotransferase (ALT), alkaline phosphatase (ALP), gamma-glutamyl transferase (GGT), total protein, albumin, total bilirubin, and direct–indirect bilirubin are conducted. In addition, HBV DNA testing and upper abdominal ultrasonography are performed. If the presence of Hepatitis D virus co-infection is unknown, screening is undertaken in this regard. If the patient is treatment-naive, initiation of antiviral therapy for HBV is commenced, and if already undergoing treatment, the regimen is continued unchanged. Regular follow-up assessments are conducted at six-month intervals.
2. HBsAg negative, anti-HBc total positive: In cases where the anti-HBc total is positive and anti-HBs is negative, LFTs and HBV DNA testing are conducted to evaluate occult hepatitis B. The patients with anti-HBc total and anti-HBs positivity were recorded as resolved HBV infection. Antiviral prophylaxis is recommended for patients with positive anti-HBc total, regardless of the presence or absence of anti-HBs, and those who accept this recommendation undergo antiviral treatment. As antiviral prophylaxis, tenofovir disoproxil fumarate (TDF), tenofovir alafenamide fumarate (TAF), or entecavir has been initiated either before or simultaneously with the onset of ocrelizumab. Once antiviral treatment has commenced, it has been continued throughout ocrelizumab therapy and for at least 12 months after completion of immunosuppressive therapy. All patients who accept or refuse to receive antiviral prophylaxis are followed-up every 6 months by checking LFTs, HBs Ag, anti-HBc total, anti-HBs and, when necessary, HBV DNA.

HBV reactivation is defined as follows: Seroconversion to HBsAg positive for HBsAg negative, anti-HBc total positive patients, and/or elevation in HBV DNA compared to baseline or absolute increase if the baseline is unavailable [11]. This definition implies a reactivation of HBV in individuals with a prior history of resolved infection.

## Statistical analysis

We performed statistical analyses using Statistical Package for the Social Sciences (SPSS) version 24.0 (IBM Corp., Armonk, NY, USA). Descriptive statistics were generated in numbers and percentages for categorical variables. The suitability of the data to the normal distribution was examined visually with histograms and probability graphs and statistically with the Kolmogorov–Smirnov/Shapiro–Wilk tests. For continuous variables adhering to a normal distribution, the mean and standard deviation were calculated. Alternatively, for variables not following a normal distribution, the median and interquartile range (25th–75th percentile) or, in some cases, the minimum–maximum range were utilized. Student's *t*-test or Mann–Whitney *U* test was used to compare the differences between two independent groups. The statistical significance limit was accepted as 0.05 (*p* value).

## Results

During the study period, ocrelizumab was administered to 405 patients out of the 3600 individuals comprising the MS cohort at our center. One patient was excluded due to concurrent immunosuppressive drug use, other than pulse steroid therapy, and an additional four patients were excluded due to incomplete or missing data. The mean age of the 400 patients included in the study was 50.5 ± 1.3 years, with 243 (60.8%) of them being female. The mean duration of MS diagnosis was 13.8 ± 0.7 years. The median duration of ocrelizumab treatment was 31 months (ranging from 5 to 55 months) with the patients receiving a median of 7 (4–8) cycles of ocrelizumab. HBV screening results at baseline are given in Table 1.

**Table 1:** Hepatitis B virus screening at baseline

HBV screening result			Total, *n* (%)^a^	Antiviral prophylaxis/treatment, *n* (%)^b^	Reactivation, *n* (%)^b^
HBsAg	Anti-HBc total	Anti-HBs			
Negative	Negative	Negative	279 (69.8)	–	–
Negative	Negative	Positive	63 (15.7)	–	–
Negative	Positive	Positive	48 (12.0)	43 (89.6)	2 (4.2)
Negative	Positive	Negative	8 (2.0)	6 (75.0)	1 (12.5)
Positive	Positive	Negative	2 (0.5)	2 (100.0)	–
Total			400 (100.0)	51 (12.8)	3 (0.8)

*HBV* Hepatitis B virus, *HBsAg* Hepatitis B surface antigen, *Anti-HBc* Anti Hepatitis B core, *Anti-HBs* Anti Hepatitis B surface

^a^Column percentage

^b^Row percentage

Individuals with a positive anti-HBc, 58 (14.5%), demonstrated a higher mean age (57.2 ± 10.6) in comparison to those without (49.5 ± 11.6, *p* < 0.001). There were two patients (0.5%) with chronic hepatitis B (positive for HBsAg), one of whom was already on entecavir and the other on TDF. Both patients continued their antivirals as prescribed during ocrelizumab treatment. Throughout the follow-up period, they maintained negative HBV DNA levels, and their ALT levels remained within the normal range.

Out of 56/400 (14%) patients with HBsAg-negative and anti-HBc total positive results, 49/56 (87.5%) were initiated on antiviral prophylaxis: 36 (64.3%) with TDF, 7 (12.5%) with TAF, and 6 (10.7%) with entecavir. Among the 48/56 (85.7%) patients receiving regular prophylaxis, no reactivation was observed during the follow-up period (median duration 24 months, range 6–60 months). Baseline characteristics of the patients with anti-HBc total positivity (excluding HBsAg-positive individuals) are given in Table 2.

Reactivation of HBV occurred in 2 out of 7 (28.6%) patients who did not receive antiviral prophylaxis and in one patient who did not adhere to the prophylaxis regimen. The demographic and clinical characteristics of these three patients are presented in Table 3. TDF was initiated in two patients (Patient 1 and Patient 3) who did not initially receive antiviral treatment, and regular use of TDF was recommended for the other patient (Patient 2).

Five out of seven (62.4%) patients, who had anti-HBc total positivity but did not receive antiviral prophylaxis, did not experience HBV reactivation during follow-up (four anti-HB- positive and one negative). The median duration of ocrelizumab treatment was 39 months (range 5–45 months). All patients with reactivation had anti-HBs levels below 100 mIU/mL and the median titer was 18 (ranging between 0 and 21). None of the patients with no reactivation had anti-HBs titers below 100 mIU/mL and the median titer was 202.5 (ranging between 183 and 304), significantly higher than the patients who experienced HBV reactivation (*p* = 0.034).

**Table 2:** Baseline characteristics of the patients with anti-HBc total positivity (excluding HBsAg positive individuals**)**

Patient characteristics	*n* = 56 (%)
Age, mean SD	57.8 ± 10.5
Sex (male)	25 (44.6)
Duration of MS disease (years), mean SD	15.4 ± 8.5
Ocrelizumab treatment cycles, median (IQR)	7 (4–8)
Previous DMT numbers	
0	9 (16.1)
1	22 (39.3)
> 1	25 (44.6)
Previous DMT	
Glatiramer acetate	23 (41.1)
Interferon beta	15 (26.8)
Azathioprine	15 (26.8)
Fingolimod	14 (25.0)
Teriflunomide	12 (21.4)
Natalizumab	2 (3.6)
Mitoxantrone	2 (3.6)
Methotrexate	1 (1.8)
Dimethyl fumarate	1 (1.8)
Pulse steroid therapy	50 (89.3)
Pulse steroid therapy cycles, median (IQR)	14 (3.5–23.5)
Anti-HBs titer (mIU/mL), median (IQR)	213.5 (52.2–462.5)
Anti-HBs < 100 mIU/mL or negative	28 (50.0)
Antiviral prophylaxis/therapy	49 (87.5)
Hepatitis B virus reactivation	3 (5.3)

*SD* Standard deviation, *MS* Multiple sclerosis, *IQR* Interquartile range, *DMT* Disease-modifying therapy, *Anti-HBs* Anti Hepatitis B surface

**Table 3:** Demographics and clinical features of the patients with Hepatitis B reactivation

Patient characteristics	Patient 1	Patient 2	Patient 3
Age	45	62	64
Sex	Female	Female	Female
Duration of MS disease (years)	17	13	3
Previous DMT	Glatiramer acetate, interferon beta, fingolimod, dimethyl fumarate	Interferon beta	–
Pulse steroid therapy (cycles)	38	17	13
Time interval between the initiation of ocrelizumab and HBV reactivation (months)	38	8	6
Antiviral prophylaxis before ocrelizumab	No	TDF, did not use regularly	Entecavir, never used
Screening at baseline			
HBsAg	Negative	Negative	Negative
Anti-HBc total	Positive	Positive	Positive
Anti-HBs	21	18	Negative
After reactivation			
HBV DNA, IU/ml	136.921.591	Negative	36.758.519
ALT, U/L	15	14	59
Antiviral therapy	TDF	Continued with TDF, regular use	TDF
Follow-up (HBV DNA, IU/ml)			
First month	132.387	Negative	–
3rd month	4.359	Negative	5.654
6th month	60	–	123
9th month	< 32	–	–
12th month	–		< 32

*MS* Multiple sclerosis, *DMT* Disease-modifying therapy, *HBV* Hepatitis B virus, *TDF* Tenofovir disoproxil fumarate, *HBs Ag* Hepatitis B surface antigen, *Anti HBc* Anti Hepatitis B core, *Anti HBs* Anti Hepatitis B surface, *HBV DNA* Hepatitis B virus deoxyribonuclease, *ALT* Alanine aminotransferases

## Discussion

In this study, 14.5% of individuals with multiple MS receiving ocrelizumab had positive anti-HBc antibodies, suggesting a potential risk for HBV reactivation. Out of 56 patients with HBsAg-negative and anti-HBc total positive results, 87.5% received antiviral prophylaxis, and no reactivation occurred during follow-up in those adhering to the regimen. On the other hand, reactivation occurred in two out of seven patients who did not receive prophylaxis and in one patient who did not adhere regularly. The initiation of antiviral prophylaxis to prevent HBV reactivation in people with MS using ocrelizumab is both effective and safe.

In terms of HBV seroprevalence, Turkey exhibits moderate endemicity, with a positivity rate of HBsAg at 4% and anti-HBc total positivity at 30% in adults [15]. According to European guidelines, in countries where the prevalence of HBsAg is > 2%, screening for HBV (HBsAg, anti-HBc total, and anti-HBs) is recommended before initiating immunosuppressive therapy [9]. In a study conducted in Turkey by Yenice et al. in 2002, investigating the positivity of HBV and HCV in MS patients, HBsAg positivity was reported at 1.19% in 84 patients. However, information regarding anti-HBc total positivity, patient follow-up, and whether patients were using ocrelizumab was not provided [16]. Our study represents the most extensive investigation of HBV seroprevalence in MS patients in Turkey, revealing a positivity rate of 0.5% for HBsAg and 14.5% for anti-HBc total. These findings indicate that evaluating these patients solely based on HBsAg positivity may not be appropriate. Especially in the baseline screening of anti-CD20 drugs, it is recommended to assess anti-HBc total results for each patient.

In patients with MS undergoing ocrelizumab, the initiation and regular use of antiviral prophylaxis due to anti-HBc total positivity have proven effective in preventing HBV reactivation. In our study, none of the patients receiving regular prophylaxis experienced reactivation. However, due to the limited number of patients in the group not receiving antiviral prophylaxis, the risk of reactivation in this group could not be fully assessed. Nevertheless, reactivation was observed in two out of seven patients who did not receive prophylaxis, and one patient who did not regularly use antiviral prophylaxis experienced HBV reactivation. In a multicenter study conducted with the participation of 15 centers in Italy, 893 patients using ocrelizumab, rituximab, or cladribine were followed. Among those at risk for HBV reactivation (HBsAg-negative, anti-HBc total positive), 53 patients were monitored, with 38 of them receiving ocrelizumab. Out of these, 21 patients received antiviral prophylaxis, 13 patients were monitored for HBV DNA levels every 3–6 months, and 19 patients neither received prophylaxis nor underwent HBV DNA level monitoring. Throughout an average follow-up period of 15 months, no cases of HBV reactivation were observed in patients, regardless of whether they received antiviral prophylaxis or not [17]. In Spain, a cohort of 540 patients using anti-CD20 drugs was followed for a median duration of 3.1 years. Among six patients at risk for HBV reactivation and using ocrelizumab, no cases of reactivation were observed during their follow-up [18].

The observation of HBV reactivation in three patients from our cohort, who did not receive regular antiviral prophylaxis, stands in contrast to existing literature. Notably, two of these patients displayed normal ALT levels, while one exhibited only mildly elevated ALT levels. Despite the virological and biochemical changes, all remained clinically asymptomatic. Following the initiation of TDF as an antiviral treatment, a swift and substantial reduction in HBV DNA levels was observed in two patients, indicating a positive response to the intervention. Furthermore, the third patient consistently maintained a negative viral load after achieving full compliance with TDF. These outcomes underscore the effectiveness of antiviral treatment in mitigating HBV reactivation and further emphasize the clinical significance of regular monitoring in patients undergoing treatment with ocrelizumab for MS. A previously reported case from Italy described a 60-year-old male patient with resolved HBV infection (anti-HBs: 10.02 mUI/mL) who encountered HBV reactivation six weeks after receiving the first dose of ocrelizumab. By the 13th week, HBV DNA levels had risen to 184 IU/ml, yet the patient remained asymptomatic with normal liver enzyme levels. Successful treatment with entecavir was administered to manage the reactivation [8]. In this study, two out of the three patients who experienced reactivation had anti-HBs levels below 100 mUI/mL, and one had anti-HBs negativity. In the Spanish study, which included 28 patients (22 rituximab, 6 ocrelizumab), the median anti-HBs level was determined as 105.5 mIU/L (range: 0–609.3), and no instances of reactivation were observed [18]. In the study conducted in Italy, no significant difference in reactivation was observed between patients with anti-HBs titers < 100 and > 100 mUI/mL. However, it was recommended to prioritize prophylaxis for those with anti-HBs levels < 100 mUI/mL [17]. In light of this information, initiating antiviral prophylaxis for patients with anti-HBc total positivity, anti-HBs negativity, or low titers (< 100 mUI/mL) is considered a safer approach. There is a need for multicenter prospective randomized controlled trials to assess the risk of HBV reactivation in patients receiving ocrelizumab for the diagnosis of MS. Despite its valuable contributions, this study is not without limitations. The single-center design introduces potential biases, necessitating validation through multicenter trials. Additionally, the retrospective nature of data collection may not capture all relevant variables, emphasizing the need for prospective studies. The median follow-up duration, while informative, may not fully elucidate long-term trends, necessitating extended monitoring for comprehensive risk assessment.

## Conclusion

This study provides critical insights into the risk of HBV reactivation in people with MS undergoing ocrelizumab treatment. The observed 14% potential risk of HBV reactivation, attributable to the presence of current or resolved HBV infection, highlights the need for vigilant monitoring and prophylactic measures, particularly in regions with moderate HBV endemicity. The effectiveness of antiviral prophylaxis in preventing reactivation in patients with resolved HBV infection underscores its clinical relevance. Our recommendation emphasizes antiviral prophylaxis, particularly for patients with low anti-HBs (< 100 mIU/mL), and pre emptive strategy for others. This study provides valuable insights that could inform guidelines on HBV prophylaxis in MS patients receiving ocrelizumab, holding implications for both national and global public health perspectives.

## Data Availability

Data are available upon request to the corresponding author.
